# Knocking down NSUN5 inhibits the development of clear cell renal cell carcinoma by inhibiting the p53 pathway

**DOI:** 10.18632/aging.204761

**Published:** 2023-06-01

**Authors:** Lei Li, Mingyang Li, Jianyi Zheng, Zeyu Li, Xiaonan Chen

**Affiliations:** 1 Department of Urology, Shengjing Hospital of China Medical University, Shenyang 110000, Liaoning, People’s Republic of China

**Keywords:** NSUN5, clear cell renal cell carcinoma, prognostic model, cell biological phenotype, p53 pathway

## Abstract

Clear cell renal cell carcinoma (ccRCC) is the most common solid renal tumor. NSUN5, a gene encoding cytosine-5 RNA methyltransferase, has rarely been reported associated with cancer. A bioinformatics analysis revealed that NSUN5 was overexpressed in ccRCC. Gene Ontology and gene set variation analyses showed that NSUN5 was associated with tumor immunity in ccRCC. The effect of immunosuppressive treatment was superior in the low-risk group compared to the high-risk group, and higher stromal score in the high-risk group relative to the low-risk group. A drug sensitivity analysis revealed that the high-risk group was more sensitive to 5-fluorouracil, mitomycin C, methotrexate, and 17-AAG, whereas the low-risk group was more sensitive to crizotinib, sorafenib, foretinib, and ivozanib. NSUN5 knockout decreased ccRCC cell proliferation. The migration speed and number of invasive cells further decreased. The percentage of apoptotic cells increased. In NSUN5-knockout cells, the levels of BAX, caspase-8, caspase-9, and p53 increased significantly, whereas those of Bcl2, CCND1, CCND3, and MMP9 decreased significantly. NSUN5 is highly expressed in ccRCC and inhibits cancer cell invasion, proliferation, and migration while promoting apoptosis by activating the p53 signaling pathway. This study provides insights into the mechanisms of action of NSUN5 in urological tumors and may contribute to improving ccRCC treatment options.

## INTRODUCTION

Among the various pathological types of renal cell carcinoma (RCC), the highest degree of malignancy occurs in clear cell renal cell carcinoma (ccRCC), which is the most common subtype. In China, the number of kidney cancer-related deaths in 2015 reached 23,400 [1]. Currently, the primary treatment methods for ccRCC include surgery, radiotherapy, and chemotherapy. However, high recurrence and metastasis rates affect patient prognoses [2, 3]. Therefore, identifying reliable biomarkers for ccRCC is critical. 5-Methylcytosine (m^5^C) is a highly concentrated epigenetic modification [4]. There are many reports on RNA m^5^C modifications, and many targets have been identified in various RNA classes and organisms [5–9]. In recent years, the methylation of multiple sites in transfer RNA has been shown to be a function of NOP2/Sun RNA methyltransferases (NSUNs) [9, 10]. The m^5^C modification of RNA has been implicated in many human diseases and tumors, including stomach and liver cancers [11, 12]. The modification and elimination of m^5^C are closely related to various cancers, and its role in tumors and clinical applications are a hot topic in research. Specific m^5^C regulators can mediate the activation of cancer pathways and provide a suitable microenvironment for tumor cell migration and metastasis [13]. For example, NSUN5 and NSUN6 have been associated with skin and breast cancer metastases [14]. However, NSUN5, in the p53 pathway in ccRCC, and its regulatory mechanisms have not been studied in urologic tumors. In our study, we first used bioinformatics to screen for high NSUN5 expression in ccRCC. In addition, we verified the expression of NSUN5 in ccRCC tissues and normal paracancerous tissues using immunohistochemistry (IHC) and detected NSUN5 mRNA expression levels in ccRCC using quantitative real-time polymerase chain reaction (qRT-PCR). Additionally, the overall survival rates of patients were compared using the TCGA database. The effects of NSUN5 on patient survival, clinicopathological characteristics, and tumor immunity were further assessed. An NSUN5-knockout ccRCC cell line was then established to observe the effects of NSUN5 on cell proliferation, migration, and apoptosis, as well as changes in p53 signaling pathway-related proteins. Finally, the tumorigenic effects of NSUN5 were verified in nude mice.

## RESULTS

### NSUN5 expression and prognostic model prediction in ccRCC

**Figure 1:**
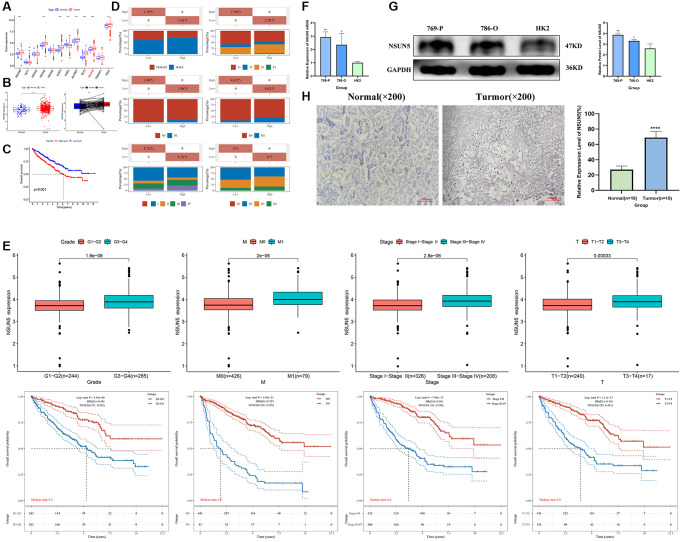
**NSUN5 expression and prognostic model prediction in ccRCC.** (**A**) The expression of 12 m^5^C methylation regulators between tumor tissues and normal controls. (**B**, **C**) Expression and survival curve of NSUN5 in ccRCC. (**D**) Relationship between NSUN5 and various clinicopathological characteristics. (**E**) The mRNA expression of NSUN5 and prognosis of grade, stage, M and T. (**F**) The expression of NSUN5 in ccRCC cell lines and HK-2 cell line. (**G**) The protein expression of NSUN5 in ccRCC cell lines and HK-2 cell line. (**H**) The protein expression of NSUN5 in ccRCC tissue and paracancerous tissue (^*^P < 0.05, ^**^P < 0.01, ^***^P < 0.001, ^****^P < 0.0001).

**Figure 2:**
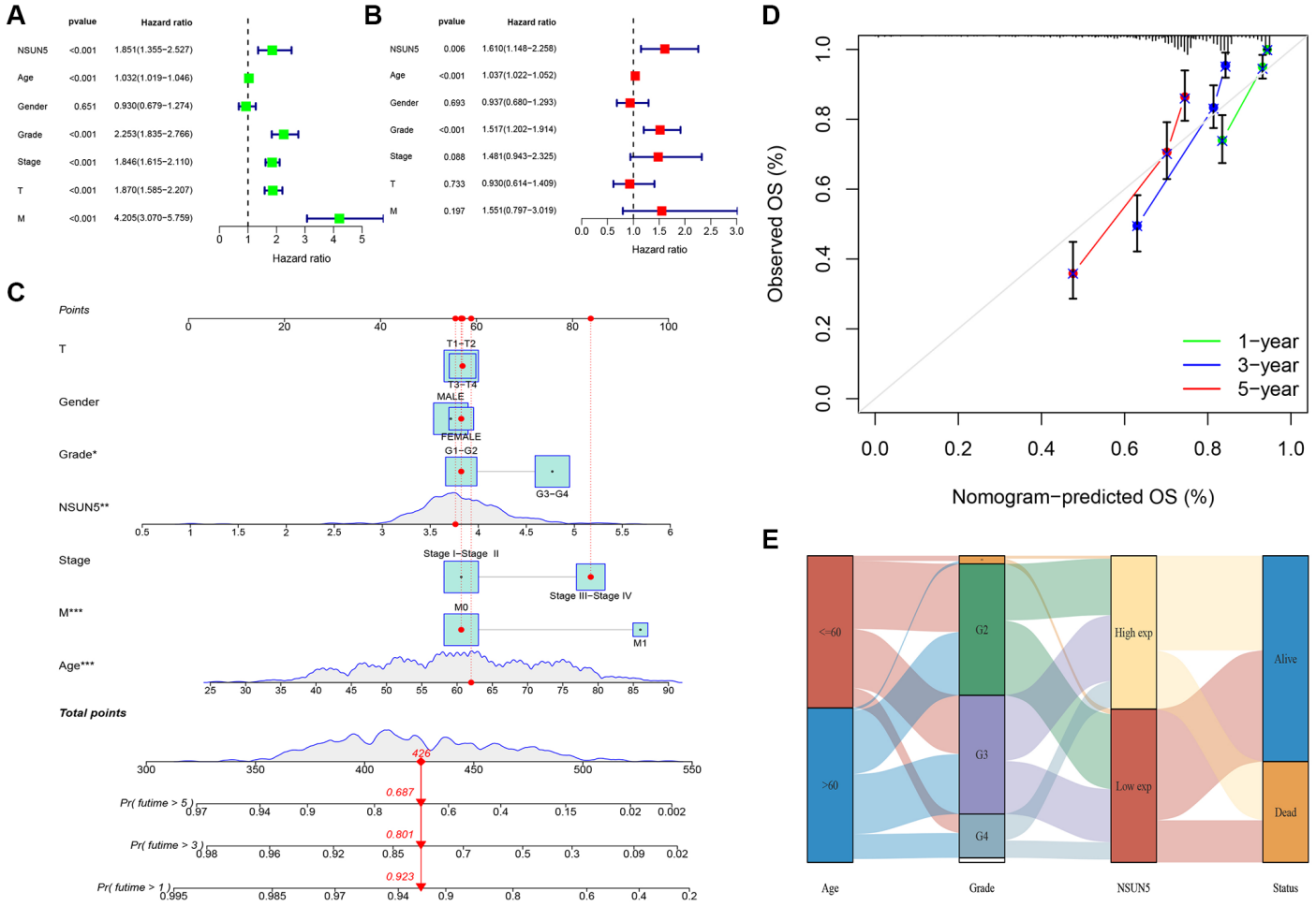
**Prognostic signature of independent prognostic factors for KIRC OS.** (**A**, **B**) Forrest plot of the independent prognostic factors in KIRC. (**C**) The nomogram of the risk model for predicting the OS probability of ccRCC patients. The whole points projected on the bottom scales indicate the likelihood of 1-, 3-, and 5-year OS. (**D**) The calibration plot for the nomogram predicting 1-, 3-, and 5-year OS. (**E**) Sankey Diagram of age, grade, NSUN5, and clinical outcomes.

TCGA transcriptome data on the expression of RNA methylation regulators in ccRCC showed that NSUN5 expression was significantly higher in ccRCC than in normal samples (*P* < 0.001) and was correlated with poor prognoses (Figure 1A–1C). The clinical factor analysis showed that high NSUN5 levels corresponded to poor clinical factors, such as metastasis and TNM stage (Figure 1D; Table 1). High NSUN5 expression was correlated with clinical features of advanced-stage ccRCC (Figure 1E). We further explored the prognostic value of NSUN5 expression. Univariate and multivariate analyses showed that NSUN5 expression, age, and grade were independent prognostic risk factors in ccRCC (Figure 2A, 2B). All clinical indicators were used to construct a prognostic nomogram (Figure 2C). The performance of the model was evaluated using calibration curves (Figure 2D). The Sankey diagram showed that NSUN5 expression and independent prognostic risk factors had a significant impact on patient survival (Figure 2E). RT-qPCR, western blotting, and IHC preliminarily verified the expression of NSUN5 (Figure 1F–1H). These results indicated that high NSUN5 expression is associated with poor prognoses and may cause adverse effects in ccRCC.

**Table 1:** The relationship between NSUN5 and clinical characteristics.

	**Chara**	**Low-NSUN5**	**High-NSUN5**	***P*_value**
**Status**	Alive	193	164	0.01
	Dead	73	102	
**Age**	Mean (SD)	60.8 (12.1)	60.4 (12.2)	0.673
	Median (MIN, MAX)	60 (26 90)	61 (32 88)	
**Gender**	FEMALE	106	81	0.029
	MALE	160	185	
**pT_stage**	T1	11	10	0.031
	T1a	75	66	
	T1b	68	42	
	T2	28	27	
	T2a	3	7	
	T2b	1	3	
	T3	0	5	
	T3a	51	71	
	T3b	25	27	
	T3c	2	0	
	T4	2	9	
**pN_stage**	N0	117	123	0.006
	N1	2	14	
	NX	147	129	
**pM_stage**	M0	241	200	0
	M1	23	58	
	MX	2	8	
**pTNM_stage**	I	153	113	0
	II	31	26	
	III	58	65	
	IV	24	59	
**Grade**	G1	9	5	0.021
	G2	130	98	
	G3	92	114	
	G4	30	46	
	GX	3	2	

### Functional analyses

**Figure 3:**
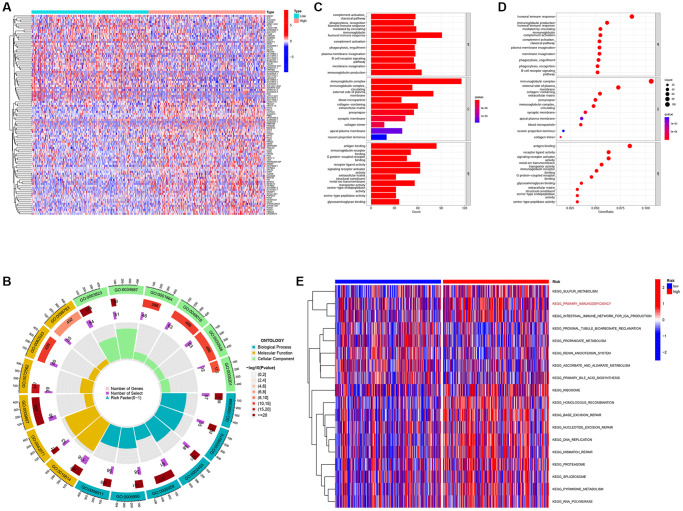
**Differentially expressed genes analysis and gene function enrichment analyses.** (**A**) Heat map showing the DEGs between the high-risk and low-risk groups. (**B**) Circle plot through Gene Ontology (GO) analysis visualizing the biological processes enriched by DEGs. (**C**, **D**) Bubble diagram and bar plot showing the signaling pathways enriched by DEGs through GO analysis. (**E**) Heatmap illustrating the result of GSVA.

To further investigate the biological processes and signaling pathways associated with risk scores, we performed a functional enrichment analysis of the DEG between the low- and high-risk groups (Figure 3A). The GO analysis showed that the DEGs were enriched in immune cell regulation and immune responses (Figure 3B–3D). In addition, the GSVA showed that immune function was significantly enriched in the high-risk group (Figure 3E). Therefore, we speculate that the DEGs identified herein are related to immune disorders in patients with ccRCC.

### Comprehensive analysis of the NSUN5 risk model and TIME characteristics

**Figure 4:**
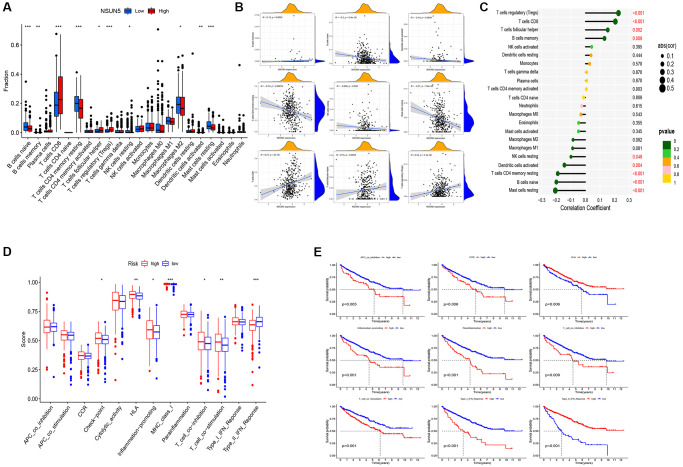
**Distinct TME characteristics of ccRCC patients according to the risk score.** (**A**) Boxplots showed abundance of 22 infiltrating immune cell types. (**B**) Correlation between nine immune cells and patient risk score. (**C**) Lollipop plot showed abundance of 22 infiltrating immune cell types. (**D**) Immune function differential analysis for single sample gene set enrichment analysis. (**E**) Kaplan-Meier curves for the 9 immune function in ccRCC patients (^*^P < 0.05, ^**^P < 0.01, ^***^P < 0.001).

**Figure 5:**
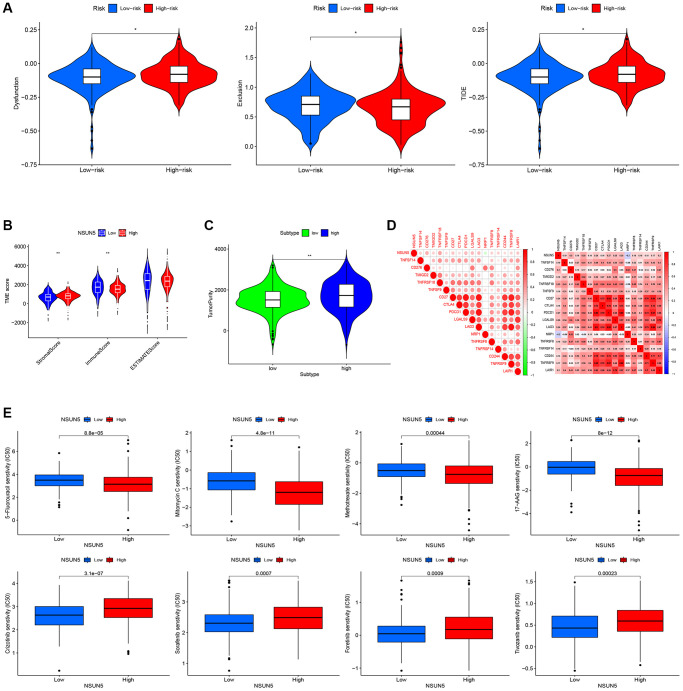
**Correlation between the risk score and the TME score, TIDE score, and immune check point.** (**A**) TIDE scores with the low- and high-risk groups. (**B**) Immune, stromal and ESTIMATE scores with the low- and high-risk groups. (**C**) Tumor purity with the low- and high-risk groups. (**D**) Heat map of the correlation between NSUN5 and immune check points. (**E**) Drug sensitivity analysis (^*^P < 0.05, ^**^P < 0.01).

We found that five infiltration enrichment fractions were significantly increased in the low-risk group: naïve B cells, CD4 cell memory resting, natural killer cells, M2 macrophages, and resting mast cells. This was in contrast to the decrease in the number of T cells, CD8 follicular T helper cells, and T regulatory cells (Tregs) (Figure 4A). The correlation between the risk group and immune-infiltrating cells is shown in Figure 4B, 4C. In addition, an analysis of the risk model and immune function showed that the high-risk group was associated with most immune functions, except for the type II interferon response (Figure 4D). The survival curve of the risk groups and immune function are shown in Figure 4E. The TIDE analysis showed the TIDE and dysfunction scores of the high-risk group were high. The results further showed that treatment with immunosuppressants was more effective in the low-risk group than in the high-risk group (Figure 5A). The TME score and immune cell infiltration analysis proved that the stromal score was high in the high-risk group, and the immune score was high in the low-risk group (Figure 5B). In addition, tumor purity was higher in the high-risk group compared to the low-risk group (Figure 5C). Therefore, it is necessary to develop novel immunotherapies for ccRCC. We further analyzed the correlations between the immune checkpoint gene signatures and NSUN5 expression and found that the expression of NSUN5 was related to that of TNFSF14 (Cor = 0.19), CD276 (Cor = 0.3), TMIGD2 (Cor = 0.18), TNFRSF18 (Cor = 0.33), TNFSF9 (Cor = 0.16), CD27 (Cor = 0.24), CTLA4 (Cor = 0.16), PDCD1 (Cor = 0.27), LGALS9 (Cor = 0.25), LAG3 (Cor = 0.25), TNFRSF8 (Cor = 0.18), TNFRSF14 (Cor = 0.18), CD244 (Cor = 0.15), and TNFR2SF9 (Cor = 0.17) and positively correlated with LAIR1 (Cor = 0.2). It was negatively linked to the expression of NRP1 (Cor = –0.2) (Figure 5D). We further assessed the effects of various drugs in the risk groups and found that the high-risk group was more sensitive to 5-fluorouracil (5-FU), mitomycin C (MMC), methotrexate, and 17-AAG, whereas the low-risk group was more sensitive to crizotinib, sorafenib, foretinib, and ivozanib (Figure 5E).

### Enhanced NSUN5 proliferation and apoptosis in ccRCC

**Figure 6:**
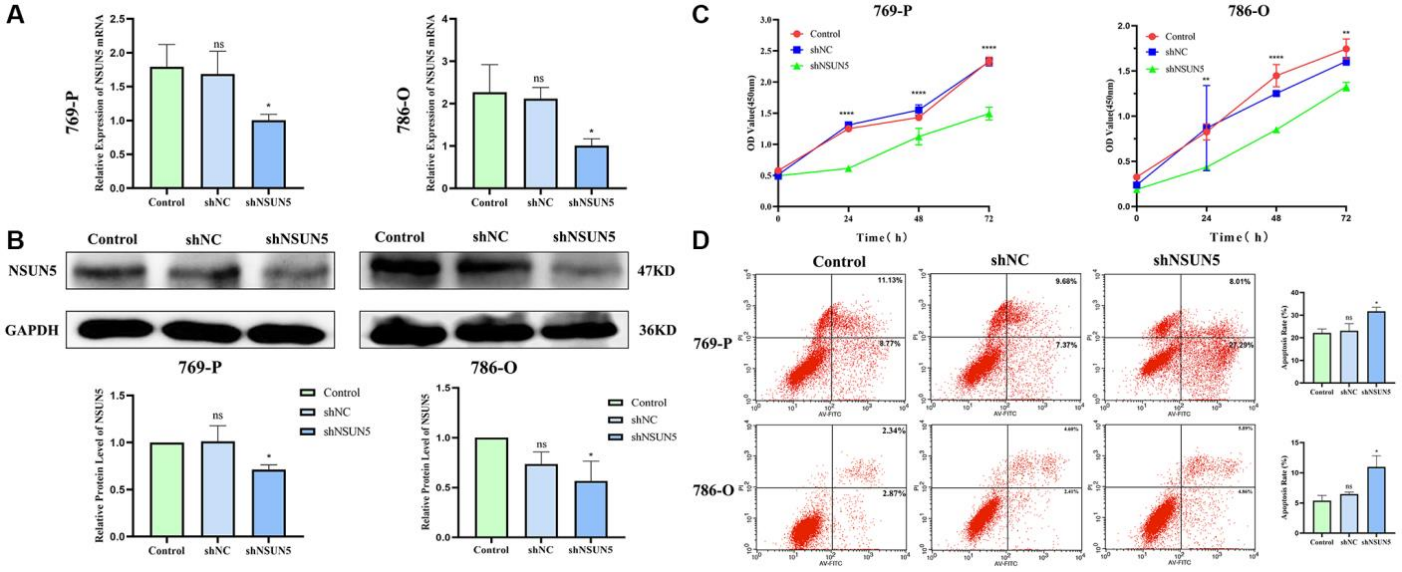
**Knockdown of NSUN5 inhibited ccRCC cell proliferation and promoting ccRCC cell apoptosis.** (**A**, **B**) Verification of knockdown efficiency of NSUN5 from mRNA level and protein level. (**C**) A CCK-8 assay was used to detect the effect of NSUN5 on the proliferation of ccRCC cells. (**D**) After knockdown of NSUN5, cell apoptosis in each group was detected by flow cytometry (^*^P < 0.05, ^**^P < 0.01, ^****^P < 0.0001).

NSUN5 short hairpin RNA was transfected into 786-O and 769-P cells. The mRNA and protein expression of NSUN5 in the 786-O and 769-P cells were significantly decreased (Figure 6A, 6B). The CCK-8 results confirmed that after NSUN5 knockout, the proliferative capacities of the 786-O and 769-P cells were significantly reduced (Figure 6C). The flow cytometry analysis proved that the apoptosis rates of the 769-P and 786-O cells significantly increased in NSUN5-knockdown group (Figure 6D).

### Wound healing assays for cell migration and transwell assays for cell invasion after knocking down NSUN5

**Figure 7:**
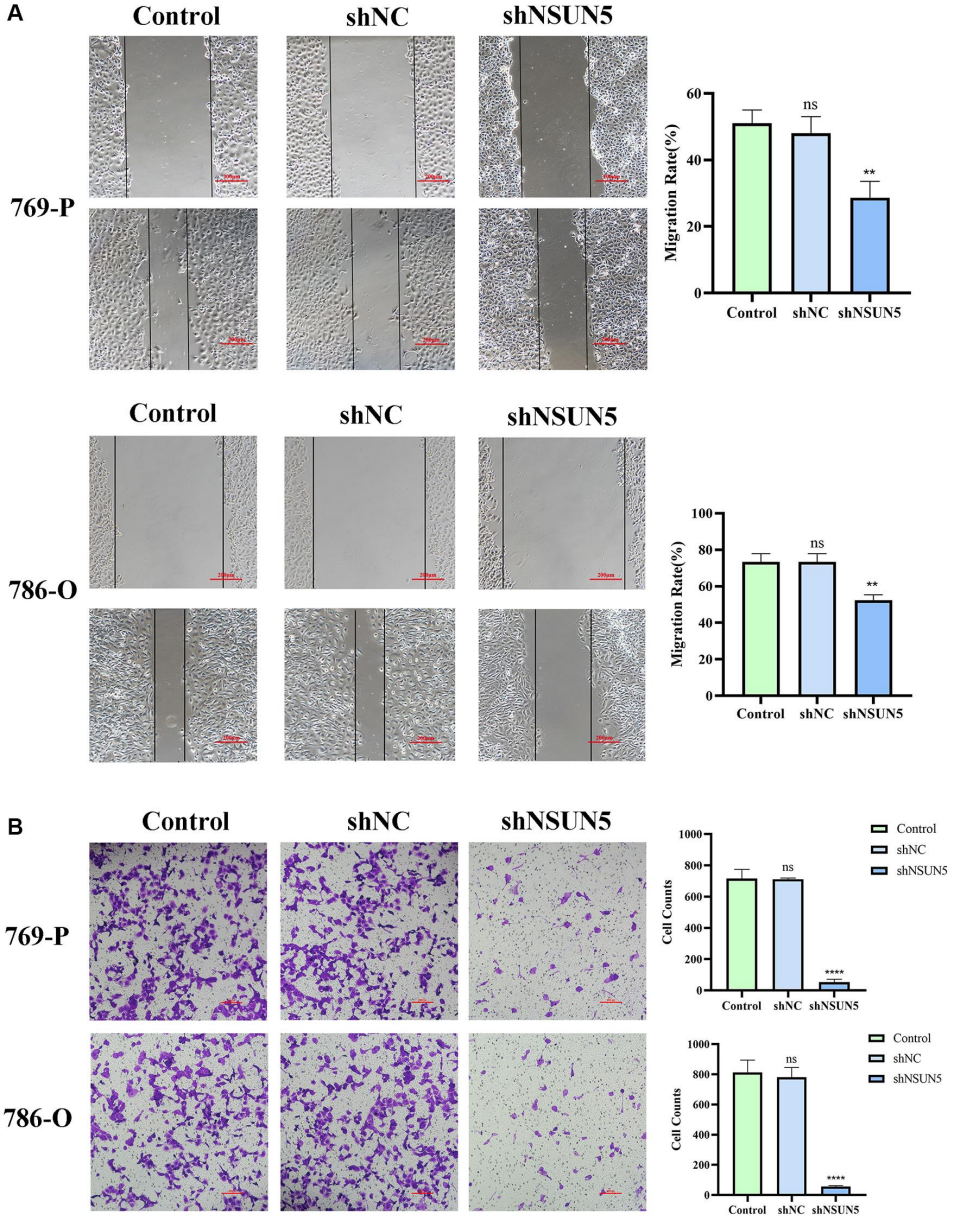
**Knockdown of NSUN5 inhibited ccRCC cell migration and invasion.** (**A**) The effect of NSUN5 on the migration of ccRCC cells was detected by wound healing assay. (**B**) Transwell assays were used to detect the effect of NSUN5 on the invasion of ccRCC cells (Observation under 200x). Data from one representative experiment is presented as Mean ± SEM (^**^P < 0.01, ^****^P < 0.0001).

As migration and invasion are characteristics of renal cancer, wound healing and transwell assays were used to validate the effects of NSUN5 on ccRCC cells. The wound healing assay showed that the cell migration rate of the shNSUN5 group was slower than that of the shNC group (Figure 7A). Tumor migration decreased after the downregulation of NSUN5. The transwell invasion assays showed that after NSUN5 knockout and downregulation, the cell penetration capacity and invasiveness of the tumor cells decreased (Figure 7B). These results indicate that NSUN5 may act as an oncogene in ccRCC.

### The knockdown of NSUN5 regulates the p53 signaling pathway

**Figure 8:**
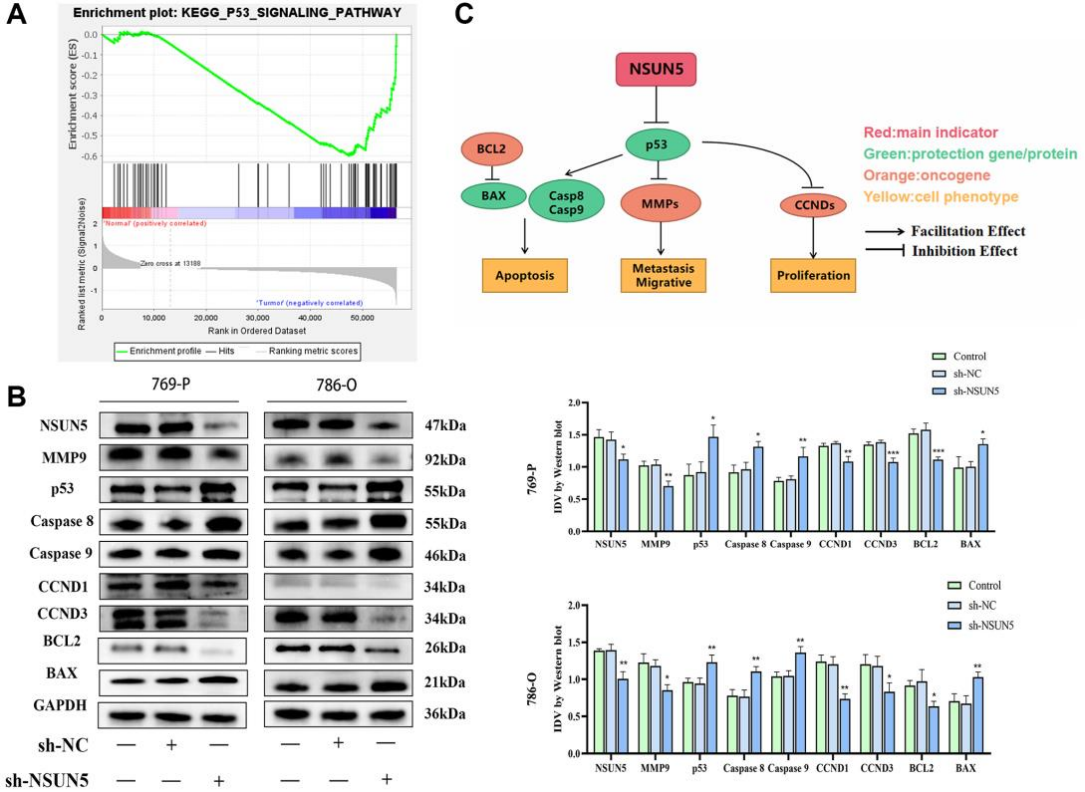
**The correlation between NSUN5 and p53 pathways.** (**A**) GSEA analysis showed NSUN5 was enriched in p53 in ccRCC. (**B**) The expression of major proteins of p53 pathway after knocking down NSUN5. (**C**) The model illustrating mechanisms of NSUN5 regulating the ccRCC via regulating the p53 pathway (^*^P < 0.05, ^**^P < 0.01, ^***^P < 0.001).

The GSEA showed that the p53 pathway was significantly enriched in the tumor group (Figure 8A). Therefore, we examined the effects of knocking down NSUN5 on p53 pathway-related proteins in 769-P and 786-O cells. NSUN5 knockdown led to the downregulation of BCL2, CCND1, CCND3, and MMP9 by activating the p53 pathway. The downregulation of NSUN5 further led to the upregulation of p53, BAX, caspase-8, caspase-9, and BAX expression (Figure 8B). NSUN5, therefore, regulates the biological behavior of ccRCC cells through the p53 pathway (Figure 8C).

### NSUN5 knockdown inhibits xenograft tumor formation

**Figure 9:**
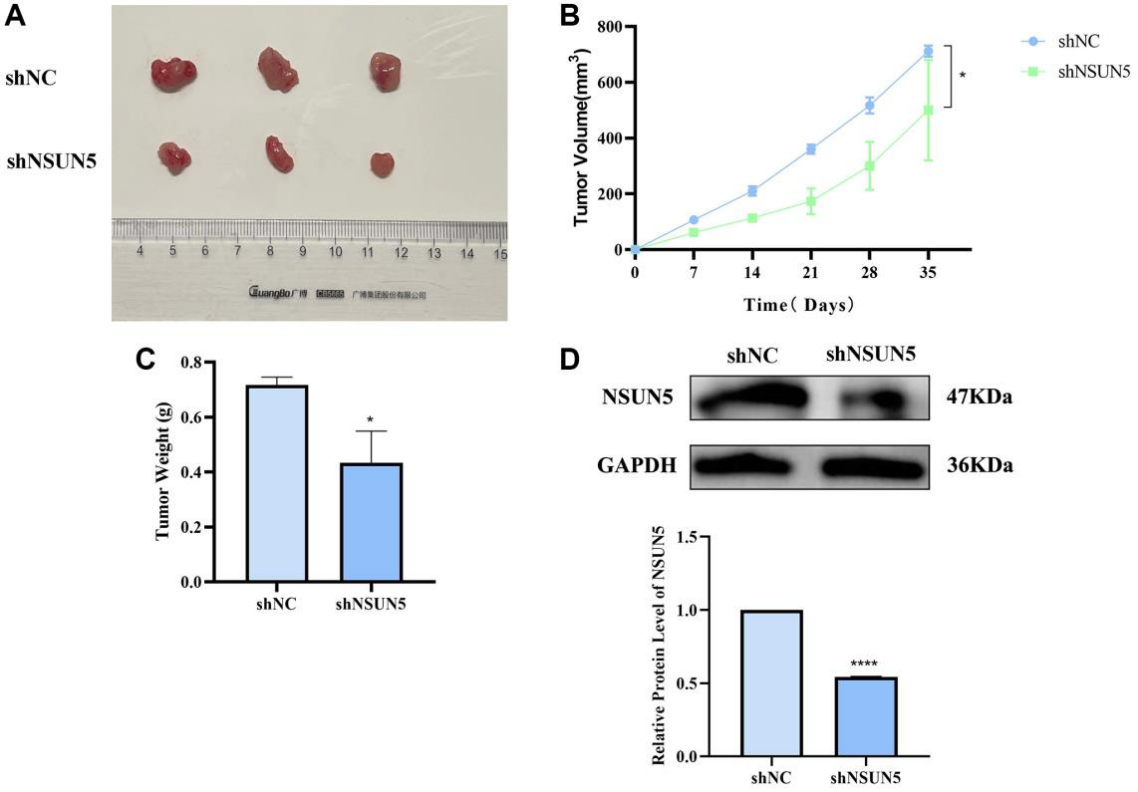
**Verify the tumorigenic effect of NSUN5 *in vivo*.** (**A**) The image of subcutaneous xenograft tumors of 786-O cells transfected with sh-NC and sh-NSUN5. (**B**) The curve of xenograft volume over time. (**C**) The comparison of tumor weight between the two groups. (**D**) Detection of NSUN5 expression in two groups of tumors. The data are shown as mean ± SD (^*^P < 0.05, ^****^P < 0.0001).

A xenogeneic ccRCC mouse model was established to study the anti-tumor effects of knocking down NSUN5 on ccRCC cells *in vivo*. NSUN5-knockdown 786-O and control cells were injected subcutaneously into mice and the volume and weight of the tumors were observed. Relative to those in the shNC group, the tumor volumes and weights in the shNSUN5 group were significantly smaller (Figure 9A–9C). The western blotting analysis further verified NSUN5 expression in the subcutaneous tumor tissues *in vivo* (Figure 9D).

## DISCUSSION

RCC has the third highest incidence of malignant tumors in the urinary and reproductive systems. More than 350,000 people worldwide are diagnosed with this disease each year [15]. Although significant progress has been made in the diagnosis and treatment of RCC, it remains one of the most common tumors. Due to its universal drug resistance and poor response to existing treatment methods, it shortens the lives of patients [16]. NSUN5 has been reported to be involved in cancer. For example, epigenetic changes in NSUN5 have been reported to predict the prognoses of patients with gliomas [5]. Yin et al. found that m^5^C methylation in peripheral blood immune cells is a promising biomarker of colorectal cancer (CRC) [17]. Jiang et al. showed that NSUN5 regulates CRC development through the cell cycle [18]. However, there have been no published reports on NSUN5 expression in ccRCC or RCC. In the present study, we verified that NSUN5 is an oncogene in ccRCC. Based on TCGA analysis, we determined the expression pattern of NSUN5 in ccRCC and its relationship with clinical characteristics in patients. NSUN5 was found to be highly expressed in ccRCC and the late stages of tumors. The risk model constructed herein may further provide insights into the clinical treatment of ccRCC. The GO and GSVA analyses suggested that NSUN5 is closely related to the immune microenvironment of ccRCC. The high-NSUN5 group had a high matrix score, whereas the low-NSUN5 group had a high immune score. Chen et al. reported that stromal cells play a significant role in ccRCC development [19]. This conclusion is consistent with our results. In addition, the TIDE and dysfunction scores were higher in the high-risk group. Therefore, the effect of immunosuppressive treatment was superior in the low-risk group compared to the high-risk group. The correlation analysis of the immune checkpoint genes proved that the expression of NSUN5 was negatively correlated with that of NRP1. A 2019 article reported that the VEGF/NRP1 axis can inhibit tumor growth and metastasis in ccRCC [20]. We also found that CTLA4 and PDCD1 expression was positively correlated with that of NSUN5. In the exploration of small-molecule drugs, we found that 5-FU, MMC, methotrexate, and 17-AAG were more effective in high-risk patients, whereas crizotinib, sorafenib, foretinib, and nivolumab were more effective in the low-risk patients. These drugs have been studied for the treatment of many types of tumors. 5-FU has been reported to be an important component of systemic chemotherapy for palliative and adjuvant CRC [21]. Lou et al. found that MMC enhanced the effect of PD-L1 blocking in non-small cell lung cancer cells and that combined treatment with MMC and PD-L1 antibodies in *in vitro* mouse models was more beneficial to tumor development and overall survival [22]. Methotrexate nanoparticles can target, induce apoptosis in, and immobilize invasive thyroid cancer [23]. The potential anticancer effects of 17-AAG and silver graphene quantum dots have also been reported in breast cancer cells [24]. Ye et al. showed that crizotinib altered the metabolic modes of A549 non-small cell lung cancer cells and inhibited ATP production [25]. Sorafenib is a currently effective first-line therapy for patients with late-stage hepatocellular carcinoma, but drug resistance has become increasingly common [26]. Therefore, sorafenib may be a treatment option for low-risk patients. Foretinib has been found to reduce the phosphorylation of c-MET in glioblastoma multiforme cells and inhibit glioblastoma cell proliferation through G2/M cell cycle arrest and mitochondria-mediated apoptosis [27]. An increased CD4+ T cells-to-Tregs ratio after tivozanib treatment has further been reported to significantly improve the prognoses of patients with hepatocellular carcinoma [28].

In our study, NSUN5 was significantly overexpressed in 786-O and 769-P cells. This result was further verified in a human ccRCC model. After the lentiviral transfection, the NSUN5-knockdown cell lines showed evident trends in migration, invasion, proliferation, and apoptosis. After the knockdown of NSUN5, cell migration, invasion, and proliferation were inhibited, and the level of apoptosis increased. The GSEA analysis indicated that NSUN5 expression was significantly associated with the p53 pathway. Among the mutated genes associated with human cancers, TP53 is the most common. It has been reported as a tumor inhibition protein in many studies [29]. Here, we found that NSUN5 knockout led to inhibited ccRCC development by activating the p53 pathway. The knockdown of NSUN5 led to downregulated BCL2, CCND1, CCND3, and MMP9 expression in two ccRCC cell lines. It also led to upregulated p53, BAX, caspase-8, caspase-9, and BAX expression. BAX, caspase-8, and caspase-9 are used as apoptosis-promoting radicals. BAX inhibition can promote the growth of breast cancer, and progesterone induces the apoptosis of ovarian and endometrial cancer cells by activating caspase-8, calcitriol, and the caspase-9 pathway [30–32]. In contrast, the over-activation of Bcl-2 and its anti-apoptotic effects is associated with the occurrence, progression, and prognosis of cancer and contributes to the promotion of the radiation and chemical resistance of various malignant tumors [33]. The CCND family is involved in the regulation of tumor cell proliferation [34, 35], and MMP9 can degrade components of the extracellular matrix and plays a significant role in pathophysiological functions [36]. Fan et al. reported that L-theanine downregulates MMP9 expression and that Snail inhibits prostate cancer metastasis [37]. *In vitro* experiments have verified that the knockdown of NSUN5 leads to inhibited ccRCC tumorigenesis. Additionally, the expression of NSUN5 has been verified in tumors. Our results showed that NSUN5 affects the biological behavior of ccRCC cells through the p53 pathway. The downregulation of NSUN5 was found to contribute the activation of the p53 pathway, which in turn inhibited the proliferation, invasion, and migration of ccRCC cells and promoted apoptosis.

In conclusion, the carcinogenic function of NSUN5 is key in ccRCC. NSUN5 may be closely correlated with ccRCC prognoses; however, the specific molecular mechanisms underlying its influence on ccRCC require further exploration.

## METHODS

### Download the ccRCC patient information matrix

RNA sequencing and clinical data of ccRCC patients were downloaded from The Cancer Genome Atlas (TCGA) (https://portal.gdc.cancer.gov/). Of the 539 tumor samples that were downloaded, 9 had duplicate data and were deleted. The clinical data of 537 samples excluded 4 patient samples with missing follow-up data.

### Bioinformatics analysis of NSUN5

We included 12 previously described m^5^C regulators and compared their expression in tumor and normal tissues [38–40]. *P* value < 0.05 and |log2(FC)| > 1.0 indicated statistically significant results. The expression of NSUN6 and NOP2 has previously been confirmed in ccRCC [41]. Wu et al. reported that both NSUN2 and NOP2 were highly expressed in ccRCC cells and tissues [42]. Cui et al. reported high YBX1 expression in ccRCC tissues [43]. The correlation between NSUN5 expression and the clinical characteristics of patients was evaluated using the “limma” package in R. The R package “ggplot2” was then used to plot survival curves. A multivariate COX regression analysis in the “forestplot” R package was used to identify independent prognostic factors. Clinical indicators and NSUN5 expression were used to construct a prognostic nomogram model through the “rms” package in R. The R package “ggalluvial” was then used to build a Sankey diagram. Next, we performed a Gene Ontology (GO) analysis for the differentially expressed genes (DEGs). Data from the gene set c2.cp.kegg.v7.4 were used to perform a gene set variation analysis (GSVA) and generate a heatmap. The Estimate and CIBERSORT algorithms were used to obtain TIME scores and immune cell infiltration profiles, respectively. Tumor immune dysfunction and exclusion (TIDE) data were downloaded from http://tide.dfci.harvard.edu/. The immune checkpoint gene sets that were obtained are listed in Supplementary Table 1. GSEA 3.0 software was used to perform a gene set enrichment analysis (GSEA). The “limma”, “ggplot2”, and “corrplot” packages were used to explore the correlations between risk groups and immune checkpoints.

### Clinical specimens and immunohistochemistry

Surgically excised ccRCC tissue samples served as the observation group, and adjacent tissues served as the control group. The paraffin-embedded tissues were cut to a thickness of 3.5 μm and incubated for 30 min after being dewaxed, hydrated, and treated with an endogenous peroxidase blocker. The tissue sections and primary antibodies were incubated overnight at 4°C. They were then incubated with the corresponding secondary antibodies for 2 h. Finally, the sections were stained with DAB.

### Cell culture

The human ccRCC (769-P and 786-O) and human renal tubular epithelial (HK2) cell lines were purchased from the Chinese Academy of Sciences Cell Bank. The cells were cultured in RPMI-1640 medium (KeyGEN Biotech, Inc., Nanjing, China) and Dulbecco’s Modified Eagle Medium supplemented with 10% fetal bovine serum (Shanghai Biotech, China) and 5% carbon dioxide at 37°C.

### Lentivirus construction and infection

Hanbio Tech (Shanghai, China) constructed a lentivirus that knocked out NSUN5. In a six-well plate, 50% of the fused cells were seeded and infected with NSUN5-knockdown lentivirus (shNSUN5), a negative control (NC), or blank control (control). Two weeks after the puromycin (4 μg/mL) transfection, stably transfected cells were obtained (the lentivirus transfection sequence is shown in Supplementary Table 2).

### Real-time polymerase chain reaction

qRT-PCR was used to verify the mRNA expression of NSUN5. Extraction of total RNA from cells was done using TRIzol reagent (TaKaRa Bio Inc., Japan). The PrimeScript RT kit was used to synthesize cDNA (TaKaRa, Shiga, Japan). qRT-PCR was performed using the 7500 Real-Time PCR System. The 2^–ΔΔ^ Ct algorithm was used to evaluate the changes in the expression of the prognostic genes relative to that of β-actin. The primer sequences for NSUN5 were as follows: forward, 5′-TGCCTCGATTTGTGCGTGTG-3′; reverse, 5′-GACAGCTGGCCCTGTCCT-3′.

### Cell counting kit-8 (CCK-8)

Cell proliferation was detected using a CCK-8 assay. A total of 3 × 10^4^ cells/mL of 786-O and 769-P cells were inoculated into each well of a 96-well plate and cultured in the dark at 37°C for 2 h. Absorbance was then measured at 450 nm. This procedure was repeated at 0, 24, 48, and 72 h.

### Wound healing assay

We evaluated the migration of the ccRCC cells using a wound-healing assay. The transfected cells were transferred to a six-well plate. The degree of cell fusion exceeded 90%, after which the cells were cut open with the tip of a 200-μL pipette to keep their edges neat and photographed under a microscope. The cells were then cultured in a serum-free medium for 24 h, and images were captured to observe wound healing.

### Transwell invasion assay

A cell invasion assay was performed using Matrigel, in which 200 μL of medium with 10% serum and 3 × 10^4^ cells were added to the upper chamber, and 500 μL of medium with 20% serum were added to the lower chamber. After 24 hours of incubation, 4% paraformaldehyde was used to fix the cells and 0.1% crystalline violet was used for staining. Finally, images were captured using an inverted microscope.

### Flow cytometry assay

Apoptosis was detected using the Annexin V-FITC/PI kit. The ccRCC cells were digested with trypsin, collected, and washed with phosphate-buffered saline (PBS). Annexin V-FITC (3 μL) and PI staining (3 μL) were then applied to the cells. After 15 min, the apoptosis rate was determined by flow cytometry.

### Western blotting

RIPA lysate and a BCA kit were used to extract the ccRCC cell proteins and determine their concentration, respectively. Sodium dodecyl sulfate-polyacrylamide gel electrophoresis (10%) was then performed. The proteins were transferred from the gel to a polyvinylidene difluoride membrane, which was blocked with 5% skim milk for 2 h. Incubation of primary antibody with membrane was done overnight, followed by incubation of secondary antibody with membrane for 2 hours. Finally, an enhanced chemiluminescence kit was used to detect the antibody signals. The following primary antibodies were used: NSUN5 (1:5000), Bcl2 (1:2000), BAX (1:2000), CCND3 (1:3000), p53 (1:2000), caspase-8 (1:2000), caspase-9 (1:2000), MMP9 (1:2000), CCND1 (1:2000), and GAPDH (1:5000) (Beijing Bioss, China).

### Mouse xenograft models and subcutaneously implanted tumors

Male BALB/c nude mice (4–6 weeks old; 18–20 g each) were purchased from the Shanghai Experimental Animal Center (Shanghai, China). All of the mice were housed in a pathogen-free environment. Six mice were divided into two groups: shNC and shNSUN5. The right sides of the mice were subcutaneously injected with 786-O ccRCC cells at a density of 7 × 10^6^ cells/200 μL of PBS to establish nude mouse xenograft models. Every seven days, the lengths and widths of the tumors were measured using calipers. The volumes of the tumors were calculated as follows: (length × width^2^)/2. After the experiment, the mice were euthanized, and the tumors were weighed. The mean volumes of the tumors were plotted at each time point (*n* = 3 per group).

### Statistical analysis

The experimental data analysis and statistical graphing were performed using the GraphPad Prism 9 software. The distribution of variables is expressed as mean ± standard error of the mean. Data from two or more groups were compared using *t*-tests or one-way ANOVA. *P* < 0.05 indicates that the results are statistically significant.

## Supplementary Materials

Supplementary Tables
