# Supplementary material for: Knocking down NSUN5 inhibits the development of clear cell renal cell carcinoma by inhibiting the p53 pathway
Source: Aging (Albany NY). 2023 Jun 1;15(11):4757–73. doi: 10.18632/aging.204761 (PMC10292888; doi:10.18632/aging.204761)
Supplement: Supplementary Tables [file aging-15-204761-s001.pdf]

SUPPLEMENTARY TABLES

Supplementary Table 1. Immune checkpoint gene set.

Symbol	Description
IDO1	Indoleamine 2,3-Dioxygenase 1
LAG3	Lymphocyte Activating 3
CTLA4	Cytotoxic T-Lymphocyte Associated Protein 4
TNFRSF9	TNF Receptor Superfamily Member 9
ICOS	Inducible T Cell Costimulator
CD80	CD80 Molecule
PDCD1LG2	Programmed Cell Death 1 Ligand 2
TIGIT	T Cell Immunoreceptor with Ig and ITIM Domains
CD70	CD70 Molecule
TNFSF9	TNF Superfamily Member 9
ICOSLG	Inducible T Cell Costimulator Ligand
KIR3DL1	Killer Cell Immunoglobulin Like Receptor, Three Ig Domains and Long Cytoplasmic Tail 1
CD86	CD86 Molecule
PDCD1	Programmed Cell Death 1
LAIR1	Leukocyte Associated Immunoglobulin Like Receptor 1
TNFRSF8	TNF Receptor Superfamily Member 8
TNFSF15	TNF Superfamily Member 15
TNFRSF14	TNF Receptor Superfamily Member 14
IDO2	Indoleamine 2,3-Dioxygenase 2
CD276	CD276 Molecule
CD40	CD40 Molecule
TNFRSF4	TNF Receptor Superfamily Member 4
HHLA2	HERV-H LTR-Associating 2
CD244	CD244 Molecule
CD274	CD274 Molecule
HAVCR2	Hepatitis A Virus Cellular Receptor 2
CD27	CD27 Molecule
BTLA	B And T Lymphocyte Associated
LGALS9	Galectin 9
TMIGD2	Transmembrane and Immunoglobulin Domain Containing 2
CD28	CD28 Molecule
CD48	CD48 Molecule
TNFRSF25	TNF Receptor Superfamily Member 25
CD40LG	CD40 Ligand
ADORA2A	Adenosine A2a Receptor
VTCN1	V-Set Domain Containing T Cell Activation Inhibitor 1
CD160	CD160 Molecule
CD44	CD44 Molecule
TNFSF18	TNF Superfamily Member 18
TNFRSF18	TNF Receptor Superfamily Member 18
BTNL2	Butyrophilin Like 2
C10orf54	V-Set Immunoregulatory Receptor
CD200R1	CD200 Receptor 1
TNFSF4	TNF Superfamily Member 4

CD200	CD244 Molecule
NRP1	Neuropilin 1
TNFSF14	TNF Superfamily Member 14

Supplementary Table 2. Lentivirus sequence of NSUN5.

Name	Detailed sequence
sh-NC	Sense: GATCCGTTCTCCGAACGTGTCACGTAATTCAAGAGATTACGTGACACGTTCGGAGAATTTTTTC
	Antisense: AATTGAAAAAATTCTCCGAACGTGTCACGTAATCTCTTGAATTACGTGACACGTTCGGAGAACG
sh-NSUN5	Sense: GATCCGCCAAGGGAAGATCTTTGCCTTCTCGAGAAGGCAAAGATCTTCCCTTGGTTTTTTG
	Antisense: AATTCAAAAAACCAAGGGAAGATCTTTGCCTTCTCGAGAAGGCAAAGATCTTCCCTTGGCG
